# Congenital heart defect specific volumetric data in children with Hypoplastic Left Heart Syndrome measured by CMR

**DOI:** 10.1186/1532-429X-13-S1-P210

**Published:** 2011-02-02

**Authors:** Christopher Hart, Eileen Pardun, Inga Voges, Dominik Gabbert, Michael Jerosch-Herold, Traudel Hansen, Hans-Heiner Kramer, Carsten Rickers

**Affiliations:** 1 Universitätsklinikum Schleswig Holstein, Campus Kiel, Kiel, Germany; 2 Brigham and Women's Hospital, Harvard Medical School, Boston, MA, USA

## Background

For each congenital heart defect (CHD) knowledge of morphological and functional variations is crucial in order to assess an individual case and initiate specific therapy, if necessary. Specific data are widely lacking. CMR-based values exist only for left ventricular (LV) and right ventricular (RV) volumes of children and adults with normal anatomy. Therefore, we sought to provide values for RV volumes, mass and function in children with Hypoplastic Left Heart Syndrome (HLHS) in order to be able to consider the defect specific variation.

## Methods

57 children (5,5±2,8 years) with HLHS were evaluated after surgical completion of Fontan-Circulation in a single center. Children with hemodynamic relevant lesions such as tricuspid- or neo-aortic-regurgitation as well as aortic stenosis were excluded. All examinations were performed in freely breathing deeply-sedated children. RV volumes and mass were measured in the end-systolic and end-diastolic phases on fast field echo cine images (TR/TE/α=1,1/1,6/60; FOV: 240x260) acquired in a short axis planes as a stack of at least 10 contiguous, parallel slices covering the full length of the ventricle.

## Results

Average volumetric data (mean±SD) are summarized in the table below. The end-systolic (ESV), end-diastolic (EDV) and stroke (SV) volume, as well as the myocardial mass (MM) were indexed to body surface area. No significant gender specific difference was found in young HLHS patients.

**Table 1:** 

**RV-EDV** ml/m^2^	**RV-ESV** ml/m^2^	**RV-SV** ml/m^2^	**RV-EF** %	**CI** l/min/m^2^	**RV-MM** g/m^2^
**73 ± 23**	**38 ± 17**	**38 ± 11**	**52,0 ± 9,8**	**3,0 ± 1,0**	**73 ± 26**

Compared to normal values from healthy children drawn from the literature and from our own data base, children with HLHS have a higher myocardial mass (p<0,0001) and larger end-systolic volume of the systemic ventricle (p=0,0013). All remaining parameters, especially the end-diastolic volume (p=0,04), ejection fraction (EF, p<0,0001) and the cardiac index (CI, p<0,0001), are significantly smaller. Intra- and inter-observer variability of the measurements was in the range of 4% and 9% respectively.

## Conclusion

CMR data on ventricular volumes and their variability specific to patients with HLHS have excellent inter-observer variability and fill an important void in the current literature. These reference values allow evaluation of an individual patient to assess his congenital heart defect. Additionally, the data can serve as a basis for longitudinal studies, as the time course of systemic ventricular remodeling after surgical completition of the Fontan-Circulation has important implications for patient outcomes.

